# “They See Us As Machines:” The Experience of Recent Immigrant Women in the Low Wage Informal Labor Sector

**DOI:** 10.1371/journal.pone.0142686

**Published:** 2015-11-24

**Authors:** Bindu Panikkar, Doug Brugge, David M. Gute, Raymond R. Hyatt

**Affiliations:** 1 Rubenstein School of Environment and Natural Resources, University of Vermont, Burlington, VT, United States of America; 2 Department of Public Health and Community Medicine, Tufts University, Boston, MA, United States of America; 3 Department of Civil and Environmental Engineering, Tufts University, Medford, MA, United States of America; Public Health Agency of Barcelona, SPAIN

## Abstract

This study explores the organization of work and occupational health risk as elicited from recently immigrated women (n = 8) who have been in the US for less than three years and employed in informal work sectors such as cleaning and factory work in the greater Boston area in Massachusetts. Additional interviews (n = 8) with Community Key Informants with knowledge of this sector and representatives of temporary employment agencies in the area provides further context to the interviews conducted with recent immigrant women. These results were also compared with our immigrant occupational health survey, a large project that spawned this study. Responses from the study participants suggest health outcomes consistent with being a day-laborer scholarship, new immigrant women are especially at higher risk within these low wage informal work sectors. A difference in health experiences based on ethnicity and occupation was also observed. Low skilled temporary jobs are fashioned around meeting the job performance expectations of the employer; the worker’s needs are hardly addressed, resulting in low work standards, little worker protection and poor health outcomes. The rising prevalence of non-standard employment or informal labor sector requires that policies or labor market legislation be revised to meet the needs presented by these marginalized workers.

## Introduction

Female economic migrants are an increasing presence in urban labor markets throughout the United States and now constitute 51% of this group of workers [1–2]. Even if women constitute more than half of the working immigrant population, there has been very little research on female immigrant workers compared to that of male immigrant workers [3–5]. Only recently have researchers started to address the links between gender and immigration. Much of that research has come from immigration studies and feminist scholarship [6–7]. In the field of occupational and environmental health, much of the focus on immigrant health has been on male migrants, largely ignoring women migrant workers [5,8–12]. This is similar to the omission of women from the migration studies conducted in the 1980s and early 1990s [6].

There is very little information on the gender characteristics of jobs held by immigrants. The US. Migration Policy report shows that the largest number of immigrant women (33%) workers are in the service sector [1,5]. Demand for women workers in domestic services, in particular, has been increasing as the number of women employed in formal economies in the developed countries is rising. The jobs now most available to recent immigrant women are domestic work, sales occupations, and light production work that are largely unskilled [1,5,13].

This form of “low wage informal labor structure,” leads to precarious and inconsistent jobs with atypical work hiring practices, contracts, and structure that are largely set by the employer with little oversight of unions or national labor laws. These companies provide competitive, and low fee services to their clients, often at the expense of the workers who are paid low wages, few or no work benefits, and job stability [14–18]. Migrant women workers in these jobs are afforded fewer opportunities for advancement and for securing the promise of economic and upward mobility [19].

Few occupational health studies have been conducted among recent immigrant women in the precarious informal labor markets such as cleaning and low skilled factory work. The current study provides a glimpse into this sector. While the occupational experiences of cleaners exists in literature, the experience of temporary women factory workers engaged in informal work is largely unknown. Occupational health studies conducted among women household service workers identified deleterious health effects ranging from burning in the eyes and throat, watery red eyes, breathing difficulty, skin burns and irritation due to chemical exposures; back injury, lack of vitality and fatigue from their intensely physically demanding jobs; and stress, sleep deprivation and depression from psychosocial stressors [12,20–26]. Research also shows that the probability of not receiving Workers’ Compensation coverage was higher among women, new immigrants, and part-time or temporary workers [27–28].

Other research focusing on precarious occupations and temporary workers shows poor working conditions replete with exposures to vibration; excessive and loud noise and the repetitive performance of defined tasks. These jobs are often associated with fatigue, back pain and musculoskeletal injury [20–21]. Precarious jobs have also been associated with increased rates of injury and chronic disease from exposure to a variety of chemical hazards as well as from long work hours and few breaks [28]. Social hazards such as workplace abuse, harassment, work insecurity, high work pressure, high emotional demands, underpayment (refusing to pay the agreed amount) and discrimination are also common [20,29] among new immigrants workers [12,30]. The reality of job insecurity, as the participants in this study face, has been associated with both poor psychological and somatic health [31–33].

The extent of work-related hazards and health risks specific to recent immigrant women in informal jobs can be deduced to be high from the existing research. However the full impact of immigrant women working in unstable, temporary low-skilled employment is largely unknown. Significant differences may also exist between male and female migrant workers in temporary employment due to the differences in their patterns of migration, job seeking practices, forms of employment available, work experiences, and health status [34–37]. The research reported here explored the nexus between gender, recent immigration, participation in low-skilled occupations and the resulting health status of the workers.

## Methods

### Data Collection

We conducted 8 in-depth interviews with immigrant women workers, and interviewed the same women multiple times (2–3 times depending on their availability). We approached ten recently immigrated women (less than three years of tenure in the United States) employed in non-traditional and insecure forms of employment from diverse backgrounds, and to capture job seeking patterns and occupational health outcomes present in such a population. Eight of the ten women participated in the study.

We collaborated closely with community-based organizations to conduct these interviews. The community participants referred the participants who were willing to be interviewed and we followed up with them with a phone call to set up the interview at which point a detailed informed consent was sought. An oral consent in the appropriate language was read to the participants. Respondents were asked if they were willing to participate and if the conversation could be audio recorded. The interviewer recorded the consent for both the interview and the audio recording on the IRB approved oral consent form. A written consent was not required to avoid leaving a written trail or recording their names, as it may have been percieved as a risk by participants. The author, in conjunction with a trusted community partner interpreted and, performed all the interviews in a language of participant’s choice. The interviews were conducted in Portuguese and Spanish and translated to English by the community partner who is adept in these languages. The interviews conducted ranged in length between 1–3 hours. Follow-up interviews were performed with all participants to record the changing dimensions in their work experiences. The interviews were conducted in an informal, semi-structured, conversational style. The interviews were held at locations deemed to be comfortable and acceptable to the participants and where confidentiality could be ensured. All interviews were conducted between 2007 and 2008. Immigrant women who participated in the study were compensated with a payment of $50. No names or documentation status were recorded. All of the study participants were (18+) adults.

We review additional in-depth interviews conducted with 8 Key informants from immigrant community groups and temporary employment agency staff, and union representatives who were broadly knowledgeable of informal working systems in the Boston metropolitan area to provide additional context for the synthesis of the data we collected from immigrant women interviews. The community organizations included immigrant churches and agencies that provide a range of immigrant services. The lead author conducted all the Key Informant interviews in English at their offices. All the 8 participants signed a written informed consent. The lead author also conducted site observations of temporary agency sites of the women day labor pickup site and a thermoplastic molding company. The Key Informant interviews are intended to provide independent background on the milieu within which the Women Day Laborer study participants worked.

The Women Day Laborer experiences were also compared to a contemporaneous immigrant occupational health survey conducted by the author among self-identified immigrant workers living or working in Somerville, Massachusetts [13]. The survey comprised of 212 self identified immigrants who live or work in Somerville, MA as cashier/baggers, cleaners, construction workers, factory workers, or food service workers of which 49.5% were female. However, women dominated the cleaning jobs (84%) and factory work (58%) in the survey.

The interview protocol and survey instruments and all study procedures for both the Women Day Laborers and Key Informant interviews were reviewed and approved by the Tufts University Social, Behavioral, and Educational Institutional Review Board (IRB).

### Data Analysis

We conducted a systematic hierarchical thematic analysis to label and categorize the data. Themes identified for the study was articulated directly by the participants or identified by the study team or were informed by the theoretical frameworks of precarious employment experiences and health consequences [31] and the vulnerable population conceptual model [38–39]. We thus drew from the conceptual traditions of both sociology and occupational health in defining and exploring health disparities for these Women Day Laborers.

Precarious employment is defined as job situations that feature atypical work contracts, with limited social benefits, entitlements, job security, and also are characterized by sporadic tenure, poor earnings and working conditions with the net result being elevated risks of ill health [35]. The vulnerable population conceptual model postulates that society, as a whole, plays a part in the assurance of health, justice and human rights for the individual. Vulnerable populations are groups that lack sufficient resources and are at risk for increased morbidity and mortality [31,38]. These models have been previously been applied to immigrant workers.

Based upon a consideration of these theoretical models a priori coding categories as detailed in Table 1 were determined and populated through a thematic analysis. The primary author coded all interview content and conducted the thematic analysis in partnership with the co-authors. Each coding category we used contained grouped narrative responses that capture the essence of each concept or theme. Our final step was to identify patterns among the codes and themes in the data.

**Table 1:** Framework of coding structures, which reflect the work-related experiences and health of immigrant women in informal work sectors.

Constructs	Concepts	Narratives
Occupational Attributes	Work Organization	Work routine
		Cleaning work activities
		Thermoset molding activities
		Packaging activities
		Other Work Organization
	Occupational Health Hazards	Chemical exposures in cleaning
		Other unhygienic exposures due to cleaning
		Chemical exposures in thermoset molding
		Musculoskeletal risks
		Other hazards
	Social Hazards	Work disparity
		Wage inequality
		Lack of breaks
		Work pressure
		Threats
		Other Social Hazards
	Occupational Health Services	Inadequate work and health and safety training
		Inconsistent use of personal protective equipment
		No access to health care
		No knowledge of Workers’ Compensation
Health Outcomes	Health Problems	Musculoskeletal problems
		Health problems due to chemical exposures
		Skin problems and allergies
		Psychological health problems
		Accidents and injuries
		Other health problems

This Table was adapted from the “vulnerable populations” conceptual model employed by Albarran and Nyamathi [38].

We compared the data from the women day laborers to the Key Informant interviewees and the occupational health survey data. This process was designed to allow the complexities to emerge from the Women Day Laborer interviews and be placed within an accurate context.

## Results

### Socio-Demographic Backgrounds of the Interview Respondents

The Women Day Laborers were between the ages of 30 and 52. The range of U.S. tenure of the participants was 45 days to 3 years. Six of the immigrant women in this sample were from Brazil, one from Colombia and one from Honduras. Except for one, none of the women in this sample had a college degree. None were fully proficient in English, though three of them have had some level of proficiency as a result of English as a Second Language (ESL) training.

Six of the immigrant laborers were cleaners and two women did factory work (thermoset molding and a packaging). Most of these respondents immigrated alone, while some had family or friends here. The most recently arrived were generally living with acquaintances. Two respondents said that they had no relatives in the United States. The analysis that follows focuses on the experiences of immigrant women laborers as observed by these eight recent immigrant women laborers and 8 key informants.

### Work Organization

#### Work routine

Though the study participants worked for either single or multiple employers, their work routine reflects the non-stand nature of these jobs and varied considerably depending on the job. There was no consistent time at which the cleaners and the factory workers started their day. Most workers had an early start time, in some cases as early as 6 or 7AM while those who have been in the United States longer began work on a daily basis a bit later mostly by 8AM, which is still earlier than the average start of 9:00AM in many occupations.

The start time and duration of work varied almost daily especially for cleaners, depending on the number of clients their employers had. Women cleaners worked about 8 to 9 hours a day without counting the time spent traveling from one cleaning job to another. This translates to cleaning four to five houses a day in quick succession. Working overtime is quite common in this trade. One of the cleaning woman said that she had worked as late as 11:00 PM or midnight.

The factory worker said that although her day starts with waiting for the pick up at 5:30 AM, the work itself does not begin until about 8:00 AM. At times her workday would end at 3:00PM or even as early as 11:00AM. This worker said that these shorter shifts result in not being able to rely on work hours which is particularly hard, “for the night shift, if the work starts at 8:00PM and if the material is over by midnight or 1:00AM we have to wait till the morning to be picked up.”

#### Cleaning work tasks

The cleaners in this study have cleaned a variety of locations including homes, offices, nursing homes, hotels and gyms. Some of the cleaning jobs were post-construction cleaning, after a tenant moved out, or cleaning after parties. Generally women felt that houses are a bigger job than offices, as offices do not need to be cleaned as thoroughly as houses. Cleaning is arduous involving a wide range of activities—dusting, moping, vacuuming, cleaning the bathrooms, kitchen, doing laundry, folding clothes, making beds, and at times cleaning lamps and other objects. Some also clean the outside of the houses, clear the garden, wash the windows, remove cobwebs, and clean the garages.

The cleaners found major differences in the modes of cleaning in the US than what they were used to in their country of origin such as the use of paper instead of recyclable rags; the use of strong chemicals instead of warm water and soap to do general cleaning; and the use of vacuum cleaners even on wood floors which can be easily cleaned with a broom. “I think that we carry this all the way from Brazil: The little rag for everything…so we use a rag…I noticed that the ladies stare at me and one came to me one day to say, you can use all the paper you want to use, okay!” The Brazilian cleaners found it difficult that cleaning was very chemical intensive and there is very little water used in cleaning. The cleaners also said that most of the clients believe in the products more than the skill of the worker. “I had a client that stopped to ask me why I was not using Windex to clean the windows.” Many workers also had to get used to carrying heavy vacuum cleaners around and have it be a part of their daily cleaning routine.

The most arduous tasks as described by the cleaners include cleaning the kitchen, bathroom, mopping, and making beds. Cleaning the bathrooms and kitchen exposed respondents to more chemicals and also required greater physical exertion to be on the floors at times on one’s knees. Three of the cleaners said that they do not use a mop to clean the bathrooms, but bend down to scrub the floors. Kitchen cleaning includes cleaning the refrigerator, microwave, stove, counters, floors and windows, some of which require the use of many different cleaning products. In the case of making beds, one woman said that it is more difficult for her to make beds than clean the bathroom, “Yes all those sheets, plus the covers, blankets and pillows then we need to adjust and pull in every corner…we need to go under and pull everything to fit perfectly in the mattress and the mattress is so heavy”. These tasks become especially difficult at the end of long day of cleaning.

#### Thermoset molding work tasks

The factory workers had a harder time describing the complex nature of their jobs, which involved technical features that they were not familiar with, such as in thermoset molding. While the worker does the task that they are required to do, it is quite evident that the employer has not made an effort to explain the complete nature of their job but have just explained the task that they need to repeat throughout the day. The skill provided is to keep the machine running than providing a basic understanding of making thermoset plastics. For example, the worker thought she made fiberglass though she knows that she works in a thermoset-molding factory. Her job really involves making small plastic machine parts. But the respondent had trouble explaining what parts she was making due to the fragmented nature of the task she was doing, that is, making one small part of a bigger component, “I do not know, I have asked but I do not really know, these are small pieces in the finishing area”. She continued to explain that there are a number of different machines at her place of work and at each machine the workers complete different tasks. Her main tasks were to remove molds coming out of the machine, and clean parts of those objects by precisely cutting the edges and polishing them with small sharp tools such as blades, files, and tweezers. She explained that a great deal of precision is required for these tasks as a small error could cause rejection of the part and a setback in production. “Polishing is hard to do. If the job is not polished properly the part is rejected and we get in trouble”. The production of minute medical devices that the company makes especially requires a very clean production environment to reduce defects.

During our visit to the site we observed that it was small from the outside, congested and without adequate ventilation. The location had a single shutter, which was open, but this small factory had few windows otherwise. The worker said that her work place was too crowded with four women working in a very tight space. A strong smell of chemicals was also present.

#### Packaging work tasks

The packaging jobs in our sample were performed in a huge warehouse (as reported by Key Informants as well as the Women Day Laborers). The warehouse had a door but no windows. About 60 to 80 people typically work in this warehouse. People work side by side and each are assigned a specific task, which may vary from day to day. Both the women factory workers have worked in packaging. Packing was the current job of one of the woman. Packing as a work task sounds deceptively simple even though in reality the tasks are often complicated. One of the women who worked in the plastics company said she did it for one month and quit because the job was too difficult and that supervisors put a lot of pressure on workers to complete their tasks quickly.

Women in general performed most of the packing jobs. There are some men involved, mostly to carry and transport heavy objects. The tasks briefed by the packaging worker range from cutting cardboard boxes, lining items up, packing, and the disposal of trash. The type of activity that each worker is assigned varies day by day. Some days it is packaging, other days it could be handling the boxes. Some of the cardboards are precut. This requires the pulling of the edges, folding them, forming them and finally gluing them. The cardboard is often quite thick and is used at times to pack heavy items, such as cans of food. The worker stands up to perform much of this work and the boxes are made on the floor. “You have to use your back, your arms, and be in positions that are not comfortable to you. You must bend most of the time trying to fold with your knee, your foot, and your hand. Gloves are not used in these tasks. At times after making the boxes they are put on the line on a belt and sent to another place to finish the job”. This involves taking the cans from a smaller box; re-labeling and then repacking the contents in a bigger box. Weight was an issue as there were about 50 cans in one box. The respondent complained about the difficulty in doing this task throughout the day and at a pace sufficient to keep up with the conveyor belt.

### Occupational Health Hazards at Work

#### Chemical exposures in cleaning

Cleaning jobs are highly dependent on the use of chemicals. According to the participants, they used between five to seven different cleaning compounds on a daily basis and throughout the day at work and varied according to the space or type of the objects being cleaned. The cleaning chemicals used ranged from Fantastik, Windex, Clorox, Ajax, Tilex, Pledge and Easy Off. Bathrooms and kitchen cleaning are especially chemically intensive. Clorox use was mostly heavy in the bathrooms and the kitchen and also in moldy areas and on black stains and spots. “Clorox-in the bathroom… I use it a lot but only in bathroom; in the kitchen Fantastik for counter tops, Windex for the windows, also soap, a scrubbing sponge and a little brush”. Depending on the house they are cleaning and the level of cleaning and polishing more chemicals maybe used, such as Pledge for polishing surfaces, bronze, silver etc. and Easy Off to clean stoves which accumulate grease and become stained. One woman said that one of the bathrooms that she cleaned had bronze counters on which she used Brasso, which turned her hands black since she did not have gloves. Most respondents said that Clorox, Tilex and Easy Off were the most objectionable cleaning products they used. They said that the smell of these chemicals does not come off the hands for a long time. Another cleaner said that, “I breathe those bad fumes all day through my nose, I can smell the stuff all day long.” This kind of cleaning is quite different from a weekly cleaning and exposure to chemicals once or twice a week in a household; instead these women have repeated exposure to these chemicals throughout the day, which some studies have shown to be harmful [20–26].

In addition to chemical agents these workers were also exposed to harmful and unhygienic conditions which is rarely addressed as an occupational hazard, “I stayed one day working at a nursing home and it was really bad because I needed to go all day picking up dirty clothes, and I threw up because …the smell…it was bad!”

#### Chemical exposures in factories

Neither of the workers was able to pin point the potentially hazardous substances that they work with. The thermoset plastic worker reported that the operations she did created a great deal of airborne particulate matter. “It is fiber and when we scratch it the dust comes out, it is fiberglass (plastics). We know this dust is dangerous and they give us masks”. She said that besides this “dust” (plastics) she did not work with any other chemicals. She also said that the product that she worked with, “smells really bad”, but did not equate this to volatile chemical exposures.

While the respondent did not know much about the product she was working with, we have knowledge about the wide variety of chemicals that are used in producing thermoset plastics [40]. On my visit to a different thermoset-molding factory (to protect the position of our study participant), the workers had different stations each producing different molds or parts of a piece. The process itself involved pouring liquid or powder into a mold, heating it to set temperatures, allowing the material to cure into its hardened form and taking it out and setting it for drying. Once dried, it is taken to trim, cut the edges and filed to fit the rest of the parts. These plastics can withstand heat. The heating process does not necessarily set the mold, but the chemical reaction between the specialized materials is the primary process behind curing the thermoset plastic material. Typical thermoset plastics are composed of synthetic epoxies, polyester, silicone or phenol-formaldehyde resins and polymers derived from crude oil and natural gas [40]. They may be further fabricated with a variety of additives such as colorants, plasticizers, biocides, antioxidants, flame-retardants, silica, asbestos and other fillers. Limited toxicological information is available on many of these compounds. The health hazards of the resin compounds are similar to those of the petrochemical industry. The exposures may be from vapors and dust during, loading, mixing or pelletizing maintenance operations. Overheating of the compounds, cleaning and finishing operations may further expose one to thermal decomposition materials of the polymers, solvents and adhesives [40]. The workers at the site I visited rarely wore a mask, neither was I offered a mask when visiting the facility. The smell of the operation was over powering and stuck to my clothing even after exiting that location. It was difficult to accurately identify the chemicals comprising this complex mixture of exposures. The lack of knowledge about these possibly toxic exposures and their health effects prevents the workers from taking appropriate precautions and may, thereby, adversely impact their health due to cumulative and repetitive exposure.

The woman who works at the packaging factory reported that she does not work with any chemicals. She said that there are some unpleasant smells but she could not tell what it was, “there is food, and also glue and others.” The most commonly used household industrial strength fast-acting glues are cyanoacrylates (super glues, Krazy glues), which are highly volatile compounds that can irritate the eyes, nose, and throat especially if such use is in a location that is not well ventilated. These compounds may also trigger asthma, skin conditions and flu-like symptoms [41–43]. These compounds have not yet been tested for carcinogenicity.

### Social Hazards—Work Place Pressures

#### Wage inequality

A cleaner said that for cleaning ten houses in two days her employer gets $800 and even if she (the helper) does most of the work she gets only $160. Community Key Informants noted that this is a fairly common practice, “some cleaning jobs are sold to others or sub-contracted out to other cleaning services or helpers because they have more work than what they can handle.” So even if the helper cleans the whole house, the client pays the employer and the employer pays a fraction of that amount to the helper.

Most cleaners are paid by the day or for the whole job rather than by the hour. The workers are paid based on their work but there is no fixed rate. Some contract owners pay only $60, while others pay $70 and a good rate is $85 per day. Such sums may be for as much as 10 to 11 hours of work per day. This is considered “normal.” There are more extreme cases as well. One woman said, “last week the man picked me up at 10:00 AM and I worked till 6:00 PM and he gave me only $25 dollars… but I did not say anything… It was only one house but it was a big job. I thought it was too little but did not say a word…It was frustrating, so I am not going again.” While there is a general pay for cleaning jobs such incidents were the cleaners get underpaid are not rare, their salary could get withheld also for any number of reasons for being a new employee, overlooking a certain spot, taking too long, etc.

The temping agencies on the other hand pay their employees weekly. They pay the minimum wage but in most cases travel expenses are taken out of the paycheck. One woman said that such garnishment results in receiving less than the minimum wage.

#### Lack of breaks

All the female respondents work long days often without breaks. In the case of cleaners the only time they stop is when they go from one place to the other. One worker mentioned that they mostly eat between jobs and often in the car. One of the temporary worker said that she gets a 10-minute break in the morning and a half an hour break in the afternoon for lunch. They said that they also do not get paid while they take a break. Workers are cautious about taking short personal breaks; “If you spend too much time in the bathroom they take the time out of your paycheck.” The woman who works at the packaging factory said: “we cannot even go to the bathroom because you know that they are working with a product that goes in the line, there are certain amount of boxes that need to be done at certain time. Sometimes you cannot even go because they are checking the time, how many minutes, then you don’t go and when you finally go, it hurts for having retained so long.” One of the Key Informants said that, not getting enough bathroom break time is a common problem among women as many of these women hold themselves longer than they should and self report health problems including bladder infections. A representative of the community group said that at many places, women don’t drink water because they are not allowed to go to the bathroom until their break time, or the lunchtime. They also said that at many places, they don’t have access to food, so they may experience hunger and dehydration. One of the cleaning workers said that she gets very hungry because her work is physically demanding and if she doesn’t eat, it makes her dizzy.

#### Work pressure

Many respondents report encountering work pressure and abuse which they find harder to endure than the work itself. The workers said that the employers do not care about health problems. The primary concern is getting the work done.

I started working with this Lady and she gave me only 20 minutes to clean the bathroom…and I could not finish a bathroom in 20 minutes …and she was there looking at me…she stopped working and stood by the door to stare at me… that was terrible for me because besides telling me how long I should spend doing my work… she was like a vigilante staring at me all the time…

The work pressure is so high; she wanted me to clean two more houses at the end of the day while my whole body was aching with cramps.

The factory workers also worked under constant supervision and time pressure.

They treat you differently if you never speak and you do your work…then there is no problem. If they see you talking a lot she would ask you to be quiet, don’t talk too much! Last time I got the wrong part in the side of the finished pieces and the supervisor took me to the boss. For a week he kept repeating, “check, did you check?” They check like 7 or 8 times during the day.

Horrible, because they look at workers and *they see us as machines*.

### Access to Environmental and Health Services

#### Inadequate Work and Health and Safety Training

None of the workers interviewed said that they received health and safety training. Both factory workers said that they received some generic work training but this was not the case with cleaners. Comparing these results with the of respondents to the OHS Survey for immigrant cleaners and factory workers, we see that many factory workers received work training (59%) while cleaners were less likely (48%). Less than half of factory workers and cleaners reported health and safety training (40% among cleaners and 44% among factory workers) [13]. This may also suggest that interviewees were not representative of the larger population as in the survey.

#### Use of personal protective equipment

Some cleaners mentioned use of gloves, but most did not use the gloves regularly. Only one cleaner reported using gloves on a regular basis, three said they do not use them and two used them only occasionally. Some found it hard to use gloves when the work needs to be done quickly and the work pressure is very high. “I cannot get used to it, I can’t work fast as when I am wearing gloves so I don’t wear it”. Some women used gloves only for hygienic purposes. “Some houses were clean so I did not feel the need to use the gloves”. And another said “I have fear and think …the chemical are dangerous…this one lady I went to work with she did not have gloves there because according to her the people over there were all very clean”. One woman said that her use of gloves depended on what she did. She said that she used gloves only in the bathrooms. But not all gloves are protective against all chemicals.

Respondents reported the use of masks only while cleaning tight spaces like bathrooms. One woman said that she tries to use masks when she uses products such as Easy Off but these were paper masks. No one used any respirators or protection for the knees.

The thermoset plastic worker used a plastic apron, gloves and a mask. However she found this discomforting due to nature of work and the irritation it causes from wearing especially masks. She said that nobody likes to wear masks because it gets very hot and dusty as well as itchy at work. The woman who worked at the packaging factory said that she used no protection.

#### Access to health care

None of the respondents reported having health insurance. Two women said that they have utilized free care. Contrary to what might be expected, the OHS survey show that only 57% of both the cleaners and factory workers had health insurance and 77% of the cleaners and 81% of the factory workers had access to a doctor [13]. Lack of insurance might be due to the precarious nature of these jobs as well as their recent immigrant status.

#### Knowledge of Workers’ Compensation

None of the workers knew about the Workers’ Compensation program. The Occupational Health Survey data show that 41% of factory workers and 33% of the cleaners had knowledge of Workers’ Compensation law [13]. The woman who worked at packaging said that she wants to complain but she does not know where to go as she does not know who her employer is, “we don’t know them, we don’t know who is hiring us. When you ask for something, nobody answers, everyone sends you to another person, oh no, that is not my responsibility, so we don’t know”. Another concern among workers is the loss of job: “you have no right to complain because they may say this person should not come back.”

### Health Outcomes

#### Health Problems Due to Work

The workers reported two kinds of health problems; those caused by physical exertion and stress and those directly resulting from hazards at work. The health complaints of the cleaners included dizziness, headaches, nausea, shakiness, burning eyes, skin irritation, blisters, dry hands, itchiness, headaches, shoulder pain, and back pain. All of the workers in this study talked about hazards at work and some health conditions as explained above. In the OHS survey, over half of the cleaners (52%) and close to half (47%) of the factory workers reported hazards at work. 35% of the cleaners and 27% of the factory workers reported health problems and injuries at work [13].

#### Musculoskeletal problems

Both the factory workers and cleaners reported the intensely physical nature of their work and the strains it caused on their body. The strain they reported came from lifting heavy objects, boxes in the case of factory workers and heavy vacuum cleaners and beds in the case cleaners. They also reported difficult postures assumed while performing work tasks, such as standing all day, lack of breaks, and fast paced work which has made these workers highly prone to neck, hand and leg strains, and back problems. “Basically, packaging is standing up all day, is kind of hard and it is unhealthy because I suffered from a lot of pain in my legs. I hurt my spine, my back from standing too long. I also have poor blood circulation, and burning sensations. You finish one box you get up and the back pain, I had to miss eight days of work because the pain was so strong. My boss said it was lack of vitamins, I suffer from headache from working at a stretch without any breaks, sometimes you want a break, even 5 min after working 5 hours”. Some cleaners said that their fingers get swollen, and that they were still painful in the morning. The OHS survey shows that 40% of the cleaners and 38% of the factory workers reported musculoskeletal problems [13].

#### Health problems due to chemical exposures

Some women talked about health problems related to the products they used. One woman shared an experience where a lack of training resulted in a very harmful situation.

I had a problem… I started cleaning the bathroom and started having an allergic reaction…I was using other cleaning chemicals, bleach and ammonia together… no one has showed or explained anything to me about the cleaning products or anything… I cannot read the labels you know? I was wearing contact lenses and having problems… I was feeling so bad, I gave up in the middle of the work and left.

Use of bleach and ammonia are commonly reported causes of work related asthma [22]. One worker interviewed said that she knows that she will get a headache whenever she uses a particular product. Another person said that smelling the chemicals throughout the day gives her nausea. Most of the women said their eyes burned while using strong chemicals, especially in the bathroom and feels suffocated and dizzy in restricted spaces. None of the respondents reported any chronic breathing problems or asthma.

Skin problems and allergies were also reported among cleaners, “Sometimes my hand was full of little bubbly blisters much like chicken pox because of the allergy to the products. They were everywhere… my face was swollen … the feeling was like having hot pepper in your face. I ended up with these black spots I did not have this before. And our skin becomes so dry”… Another cleaner with an allergy said “My difficulty is with people that uses Easy-Off and also Tilex…I have allergy… I had serious problems with that product before…I went from doctor to doctor and had bad allergy to that…my face was swollen and it was bad…one doctor said you are prohibited to use that product…this is killing you… my difficulty is to work with that and many people work with that…and it is hard because I need the money”. Some of these workers know the dangers of chemical exposures but find it hard to not be exposed to them.

The woman who works at the thermoplastic molding factory said that there is a lot of dust at her place and it itches, “It is something that makes the skin itch, the fiber. When I work at the machine I feel that it itches more. I know that it gets inside the skin because when I came out of work I clean my nose and white stuff comes out of my nose”. She said that she washes up a couple of times during the day because it irritates her skin. She did not report any health problems like allergies or headaches and pain. We see some similarities in the OHS survey data, cleaners reported comparable hazards at work, and higher health problems due to work than factory workers. Forty-three (43%) of the cleaners and 14% of the factory workers in the OHS survey reported allergies [13]. Close to half the cleaners (49%) and 16% of the factory workers reported chemical hazards at work in the OHS Survey [13].

#### Psychological health

Most women said that they suffered from stress, depression, excessive tiredness due to work pressures and job insecurity. One of the woman, who has been in the United States for less than two months, and stated that she was close to 50 years of age and that it is hard to work continuously without stopping throughout the day, that it is both physically, and emotionally tiring. “I started working for someone and went for 2 days and I am at home today because the work was so hard I could not go another day without resting first. I could not take it, I needed to rest”. The workers in the study also talked about sleeping difficulties, one woman said that she usually sleeps only four hours a night and at times only two hours a night. Amongst the OHS survey respondents, 40% of both cleaners and factory workers reported psychological hazards related to work.

The community Key Informants stated that most of the injuries they see among their clients are the result of lack of training and safety equipment. Further ergonomic considerations are essential in these physically demanding jobs to reduce and prevent muscular strain and cramps. Negotiation with the employers could reduce some of these risks, but many of the recent immigrants do not do that due to fear of loosing jobs, language difficulties, and perhaps because they do not know that this is something that they can discuss with their employers. Often these workers will have to use the products that their employer provides them or leave the job rather than negotiating with the employer to lower the exposure to the chemical, such as, by picking a cleaning product that is not as harmful. The work safety and health is often not a priority in these informal sector jobs that offer low fee services to clients. All the women interviewed said that they have gone to work despite feeling sick because they could not afford to take the day off as they needed the money and were afraid of losing the job. “If you (I) do not do the job correctly they might decide not to renew my employment”.

## Discussion

Though the overview of occupations in this study may seem deceptively simple at a glance, an in-depth observation reveals the complexity and arduousness of these occupations. The contingent nature of these jobs, coupled with poor work structure and agreements, low job security, poor compensations/benefits, worker training, inability to negotiate with the employer, and regulatory oversight steadily escalates the risks within these jobs [28,31–33]. Gender segregation, immigrant status, English proficiency, education and lack of documentation further relegate these workers to these temporary contingent jobs [13,44–48].

Women Day Laborers in this study reported multiple hazards. The cleaners reported that they clean for lengthy intervals and are continuously exposed to chemicals throughout the day. The type of cleaning agents used were consistent with other studies and include detergents or surfactants employed to lower surface tension of water, water softeners, pesticides, alkaline agents, solvents, corrosion inhibitors, film formers, polishes and acids to dissolve and bind calcium, regulate pH, dissolve fatty substances, and disinfection agents to kill virus, bacteria and mold [23–24]. The high risks attributable to domestic cleaning are supported by results from several studies and case reports [12,49–51]. Health concerns related to cleaning reported in this study as well as others include musculoskeletal problems due to repetitive movements (as in [52–53]), respiratory conditions (as in [22–25]), and psychosocial hazards (as in [12,51]). The static postural load in these tasks is also high [52–53].

The hazards found in factory jobs have not been fully characterized in the research literature. Some of the workers in this study knew that there were hazards associated with the commercial products with which they were working while the factory workers were generally unaware of the industrial nature of exposures at work. In addition, the health risks of many of the chemicals that the workers are exposed to are often unknown, thus preventing full understanding of the risks by these workers[54].

Marfleet & Blustein ([55], p.384) concluded that the, “US economic sector has been using the vulnerabilities of this work group to their advantage without offering adequate protection to these workers”. While these immigrants have been exploited in a host of ways, their presence is tolerated, as they are valuable to the commercial sector and the economy. Corporate or business interests play a role in the wide variety of risks and social oppression these immigrant workers endure. “At work, these migrant workers are exploited to an extent that is rarely acceptable among regular, legal employees” ([55] p 284, and [56–57]). These “underground” or informal labor markets are also largely unregulated and often outside the oversight of Occupational Safety and Health Administration (OSHA) thus making it difficult to protect the health and safety concerns among these workers. The employers are free to dismiss workers at will or with minimum resistance, as immigrant workers, and temporary workers seldom benefit from the protection of labor unions or the oversight of regulatory agencies.

With no or extremely limited enforcement of regulations, immigrant workers often quietly endure these work related problems as part of the job. Their vulnerability keeps them in their jobs thus exposing them to chemicals that can cause health problems instead of seeking protection or a safer job. To seek justice, they will have to organize themselves to get help from the human rights and worker groups in the community. Such actions are often frightening for the immigrant worker who lacks documentation status. Their need for invisibility in the society to avoid deportations, imprisonment or forced dislocation [55,58] limits the ability of these workers to seek just outcomes, and often prevents them from availing themselves of laws and regulations established to protect the rights of workers.

Governments often lack the political will to change the regulatory structure in order to protect and empower immigrant workers. Most regulations, policies and labor laws are based on standard employment relationships or upon the models of employment where a worker has a single employer, works full-time year round with full benefits and who works on the employers’ premises under his or her supervision [35]. Responses from the Women Day Laborers show that low skilled temporary jobs do not have any prior work descriptions; they are fashioned around the needs of the employer and have little ability to negotiate for the worker’s needs than other jobs. Eligibility for certain programs, such as employment insurance, work benefits and union contracts are usually based on the permanency of work or as found in standard (more mainstream and traditional) jobs and forms of employment [35,59]. Workers’ Compensation is available only to workers who work for employers who pay their premiums. Some scholars state that the Federal government supports such an, “imbalanced power system within these jobs where workers rights are ignored” [35]. The political will might be stronger if there were a large popular outcry and concern about this problem, which there is not. Also, to the extent that these workers are undocumented, the Federal government position is that they should be deported than provide justice within these highly prevalent jobs and to this large underground economy.

In order to support more effective regulation in this informal labor sector additional research is necessary. Such research must cross the chasm of race and ethnicity. For individuals with little to no control over decisions that affect their livelihood, the lack of support from government, regulatory protection, and unions leaves the immigrant worker very vulnerable to social oppression [56]. Tompa et al. [31] contend that if labor market changes are not adequately addressed, it may have long-term health, wellbeing and productivity implications for the broader society.

The development of policies, enforcement and interventions to improve worker safety and security and moving the workers out of the informal sector and into more structured employment regimes with better protection are needed to improve the quality of life enjoyed by workers employed in such informal and precarious labor markets. In non-unionized jobs like small-scale cleaning operations and factory work being part of a workers cooperative is perhaps an approach to secure such advantages [60]. Provision of language training, vocational training and other educational and social service structures are also especially important in improving economic opportunities for these workers to obtain safer and more secure employment and to advocate for improved human rights for immigrant workers. The workers’ own voices perhaps best capture and express these needs:

“No they could pay more but also treat us better…always have gloves…for us to have access to information…about the products we are using. Knowledge about the product…But you know that, all the bathroom that do not have a window have a fan. As a helper I think nothing… I think that everybody should be treated better with more justice…that they always pay well”.

“I think the first thing we should know is where are we going… know what is the work, where is the job located, how much are they going to pay us. Know the basics and what labor laws we have, in a case of an accident, or an abuse case by any one”.

By empowering the individual worker and through the formation of worker cooperatives we can perhaps avoid some of the most egregious cases of abuse [15].

The present study is primarily limited by the small sample size. The immigration status of these women, the rise in xenophobic reactions since 9/11, the increased prevalence of immigrant raids and deportations, and the existence of undocumented work status all pose real barriers to such workers openly discussing their occupational health experiences. As a result of the rapport established through the mediation of our community partners, this study does offer valuable insights into this particularly vulnerable and extremely hard to reach group of Women Day Laborers. We also provided contextual information to improve our ability to correctly identify the principal factors that shape the work experience of the Women Day Laborer respondents. Our work hopefully increases awareness regarding the deleterious experiences of newly immigrant women laborers engaged in the informal work sector. This work highlights the need for better labor market legislation and regulatory policies to ensure that these workers are adequately protected.

## Conclusion & Policy Recommendations

We detail the hazardous nature of the informal work sector and the social factors that escalate risks especially amongst recent immigrant women workers. We show that a deeper understanding of the socio-economic and political context is required to fully understand the occupational health and safety risks among Women Day Laborers. The structure of work found among low wage skilled occupations coupled with the immigrant status of women, and the lack of regulatory standards and oversight of these occupations foster oppression at work, pernicious abuses, and consequent health outcomes. The rising prevalence of non-standard employment and temporary employment requires that policies or labor market legislation be revised to meet the needs presented by these rapidly growing forms of employment.

These workers require the protection of both their individual needs and collective rights. They pose the problem in stark terms, “*They see us as machines*”. Significant change to the employment regimes and to the level of oversight and regulatory structure that these individuals receive while at work must be a first step in ameliorating the already noted disparities in occupational health and safety risks.
